# Effect of De-Icing Chemicals on Concrete Scaling: The Role of Storage Water

**DOI:** 10.3390/ma16144928

**Published:** 2023-07-10

**Authors:** Petr Misák, Dalibor Kocáb, Patrik Bayer, Tomáš Vymazal, Pavla Rovnaníková

**Affiliations:** Faculty of Civil Engineering, Brno University of Technology, Veveří 331/95, 602 00 Brno, Czech Republic; dalibor.kocab@vutbr.cz (D.K.); patrik.bayer@vutbr.cz (P.B.); tomas.vymazal@vutbr.cz (T.V.); rovnanikova.p@fce.vutbr.cz (P.R.)

**Keywords:** concrete, scaling, de-icing chemicals, water storage, statistical analysis, surface layer test

## Abstract

This paper deals with the effect of the character of the water used for the water storage of concrete test specimens on the results of tests for resistance to de-icing chemicals. Two experiments were conducted to investigate the effect of the content of free CO_2_ in water and leaching of calcium hydroxide from concrete on the test results. In the first experiment, the resistance of mortars to water and de-icing chemicals was investigated. It was found that the character of the water storage, i.e., fresh water vs. previously used water, can significantly affect the test results. The second experiment focused on investigating the effect of the content of free CO_2_ in water on the test results. It was found that the content of free CO_2_ in the water can statistically significantly influence the test results. In conclusion, the paper shows that the character of the water used for water storage of concrete test specimens and the content of free CO_2_ in water are essential factors that can significantly affect the results of concrete resistance tests to de-icing chemicals. Further research is needed to understand these influences and their potential use to improve the resistance of concrete.

## 1. Introduction

Concrete is the most widely used material in the construction industry. Indeed, it is the second most used material on Earth after water [1]. However, the production of concrete is associated with sustainability issues since it is made primarily from cement, aggregate, and water, and each component directly impacts the environment [2]. Global cement production in 2021 was approximately 4.1 billion tonnes [3], while annual concrete consumption is about seven times higher [4]. The production of Portland cement, until recently the primary component of concrete, is responsible for about 7–8% of global CO_2_ emissions [5]. This significant carbon footprint leads to growing concerns about the sustainability of concrete structures and the depletion of natural resources such as aggregate or water [6,7,8]. Sustainability assessment tools include three fundamental pillars of sustainability—economic, ecological, and socio-cultural [9]—and designing and building durable structures is closely related to at least the first two pillars.

Durability is a very general term, as it is not defined in terms of time, for example, compared to the service life of structures. When assessing the durability of a material, it is necessary to precisely define the environment in which it is to be evaluated [10]. The durability of concrete can be characterised by its ability to resist the action of external and internal agents, with the mode of failure of the concrete being highly dependent on the type and intensity of the action. The same concrete may exhibit a certain level of resistance to the action of soft (low mineralised) waters (this is corrosion caused by the leaching of calcium hydroxide; see, e.g., [11]) yet at the same time, a completely different level of resistance to the action of sulphates on concrete (this internal structural damage is caused by the increase in the volume of products of the corrosion reaction; see, e.g., [12]). One of the basic durability properties of concrete in mild climates is undoubtedly its resistance to frost exposure [13]. This refers to the ability of concrete to resist the repeated alternation of positive and negative temperatures under simultaneous exposure to water, high humidity, or water in combination with de-icing chemicals. The alternation of positive and negative temperatures has no significant adverse effect on dry concrete [14].

If a concrete structure or a part of it is exposed to water and alternating positive and negative temperatures, it is necessary to assess whether the concrete surface is also affected by any de-icing chemicals—most often by salt and the resulting NaCl solution, which may affect the concrete surface directly or in the form of salt mist or spray. When a de-icing chemical is applied to concrete, the degradation processes completely differ from those in concrete only exposed to water, freezing, and thawing. Without the action of de-icing chemicals, water will enter the pore structure of the concrete, due to absorption, and will freeze there at sub-zero temperatures. Consequently, there is a risk of micro-cracks and then cracks in the internal structure of the concrete, i.e., the concrete may become damaged. On the other hand, scaling due to salts is defined as surface damage. This is caused by frost and the resulting crystallisation of the salt solution in the surface layer of the concrete [15,16]. The concrete scaling mechanism is quite different from that of freeze–thaw, which causes water to crystallise in the internal structure of concrete, thereby causing it to fail. In the case of scaling, it is a surface action only. While this does not affect the quality of the concrete inside the structure, it makes it sensitive to the penetration of water and other aggressive agents into its structure. This reduces the overall resistance of the concrete and, consequently, the service life of the structure, as well as the risk of reinforcement depassivation. Scaling was extensively studied and described many years ago, but is still remains a subject of many papers today; see, e.g., [17,18,19,20,21,22]. The most fundamental findings in the study of concrete scaling are:

- Damage to the surface layer of concrete is significantly worse when the water applied to the concrete surface contains a certain amount of solute [23].
- The effectiveness of the concentration is almost independent of the character of the de-icing chemical in the solution (e.g., salts, urea). All these de-icing chemicals have been found to have similar effects on concrete [24].
- Damage to the surface layer of concrete is initially reflected in the formation of small chips or flakes of material that gradually fall off the surface [25].
- Without a free liquid with de-icing chemicals on the surface of the concrete, actual scaling will not occur, even when the concrete is saturated with water and subjected to alternating freeze–thaw cycles [26].
- The concentration of salts in the liquid applied to the concrete surface is more important than the concentration of the liquid in the pores of the internal structure of the concrete [23].
- The resistance of concrete to scaling is generally relatively low, but can be increased significantly by adding an air-entraining mixture to the concrete [24,27,28,29].

Testing the resistance of a concrete surface to scaling is quite difficult itself. Several standards and guidelines, both in Europe and North America, define the testing procedure differently (see Section 2). Many factors, some of which are listed above, will influence the outcome of a scaling test. At the same time, these tests are characterised by relatively high variability in the resulting values, including repeatability and reproducibility [30]. The tests deal with the length and temperature of the freeze-thaw cycles, the test specimen surface area, the characteristics of the de-icing chemicals, etc. Still, the effect of the water storage in which the test specimens are placed before the test has yet to be considered. It is reasonable that when testing a cut through a test specimen (e.g., the procedure according to standard [31]), the water in the water storage does not influence the test result. However, it is also reasonable that the surface should be tested rather than the cut through the specimen, as this is significantly more representative of the actual behaviour of the concrete in the structure. In this case, the water in which the specimens are stored before the test may have an influence.

This paper deals with this overlooked effect and presents the results of two consecutive experiments. The pilot experiment showed that the level of water saturation with calcium hydroxide in the water storage affects the results of concrete scaling tests. The second, larger experiment further refined the results of the pilot experiment and more precisely defined the conditions under which concrete scaling test results can be significantly affected.

## 2. Testing Methods

The basic concept of all methods that evaluate the resistance of concrete to the action of de-icing chemicals is similar. The test specimens to which the chemical solution is applied are placed in a device which allows automatic cycling between positive and negative temperatures. The main parameters and procedures of the various tests by which the resistance of concrete to scaling can be evaluated are briefly described below.

### 2.1. Procedure According to SS 13 72 44 [32]

This test, developed in Sweden, is called the “Borås test method” or the “slab test” [19] and is one of the basic methods. The test requires a minimum of four specimens with a tested area greater than 0.0225 m^2^. The recommended size of the specimens is 150 mm × 150 mm × 50 mm. The test is conducted one-dimensionally, the change in temperature and humidity affects only the surface to be tested, and the other specimen surfaces are insulated. Not more than 15 min before the test, a 3% NaCl solution is applied to the test surface up to a height of 3 mm. The test cycles between temperatures of −18 °C and +20 °C, with a cycle time of 24 h. The test evaluation is usually conducted after 7, 14, 21, 28, 42, and 56 cycles. The result is the cumulative amount of material scaled off the specimen in kg/m^2^ [32].

### 2.2. Procedure According to CEN/TS 12390-9 [31]

This test method is based on the standard [32] and differs only in minor details—the testing is conducted on the cutting surface of the test specimen, cycling between temperatures of −20 °C and +20 °C. This standard [31] is also the basis for the standards used in Lithuania, France, and the USA.

### 2.3. Procedure According to RILEM TC 117-FDC/CDF [33]

This test method is used in Germany. It is necessary to produce five or more specimens with a test area of more than 0.08 m^2^—for example, test cubes (150 mm × 150 mm × 150 mm). The mould for the cube production is covered with Teflon foil in the middle, and no demoulding agent may be used during the production. The surface to be tested is adjacent to the Teflon surface. The specimens are placed on pads in an automatic device into which a 3% NaCl solution is poured to submerge 5 mm of the cubes. The minimum test temperature is −20 °C and the maximum is +20 °C. One cycle takes 12 h, and the result is the cumulative amount of material that has scaled off from the specimen in kg/m^2^ after 14 and 28 cycles (4 and 6 cycles are also recommended [33].

### 2.4. ASTM C672/C672M-12 Procedure [34]

According to the American Standard [34], at least two specimens with a minimum test area of 0.045 m^2^ and a height greater than 75 mm must be tested. A 4% CaCl_2_ solution is poured on the test specimen up to a height of 6 mm (3% NaCl can also be used). The test specimens are tested by alternating temperatures between −18 °C and +23 °C; the duration of one cycle is 24 h and a visual inspection of the surface to be tested is conducted after every fifth cycle. The final evaluation is performed after 50 cycles [34].

### 2.5. Procedure According to MTO LS-412 and BNQ NQ 2621-900 [35]

The MTO LS-412 test procedure is used in Ontario, Canada. The test is performed on at least two specimens with dimensions 300 mm × 300 mm × 75 mm. A 3% NaCl solution is applied to the test surface up to a height of 6 mm, and the specimen is then placed in a device in which it is cyclically frozen at −18 °C and thawed at +25 °C. One cycle lasts 24 h, and the condition of the test area is visually assessed after every fifth cycle [35]. The BNQ test procedure is used in the province of Quebec in Canada. The minimum number of specimens to perform the test is two, with a minimum tested area of 0.05 m^2^. A 3% NaCl solution is poured onto the test specimens, which must then be covered with a waterproof membrane to prevent evaporation. The test specimens are exposed to cyclic temperature changes from −18 °C to +25 °C in an automatic test chamber for 24 h per cycle. After 7, 21, 35, and 56 cycles, the amount of the material that has scaled off from the surface of the tested area is determined, and the test result is the cumulative amount of the scaled off material from the specimen in kg/m^2^ after 56 cycles [35].

### 2.6. Procedure According to ČSN 73 1326 [36]

This test method is commonly used in the Czech Republic and a slightly modified version in Slovakia. The standard [36] lists three procedures according to which the test can be performed. Only method A (DiCh-A method) will be described in detail here, as it was used for the experiment described below.

The test is conducted on three test cubes (150 mm × 150 mm × 150 mm). After removal from the moulds, the specimens are placed in water storage at 20 °C. When the concrete is 28 days old, an automatic cycling test is started, where the upper surface of the specimen is tested without any modification. The individual test specimens are placed in test containers made of a non-corrosive material, which allows the test specimens to be immersed in a 3% NaCl solution. The material that scales off from the specimen is captured. The solution is poured into the container so that the test cubes are immersed to 5 mm around their circumference. One freeze–thaw cycle takes 2 h, the minimum temperature is −15 °C, and the maximum is +20 °C. This is a relatively quick test, at least compared to other standards. After every 25th cycle, the amount of material that has scaled off from the specimen surface in the dried state is determined. The surface resistance of cement concrete to water and de-icing chemicals is given by the amount of the scaled off material from the specimen in g/m^2^ after a certain number of cycles. More information on this method can be found, for example, in papers [37].

## 3. Experimental

Two sub-experiments were conducted as part of this study. Three laboratories participated in the first experiment, which can be called a pilot. The motivation for this experiment was the repeated failure of one of the laboratories to participate in inter-laboratory comparison proficiency testing programmes. The laboratory tried to determine the cause of long-term higher values of the amounts of scaled off material from specimens within the DiCh-A testing method. It was assumed that the reason might be the water where the concrete specimens were cured before the actual testing. This was because the laboratory cleaned the water storage tanks significantly more frequently than is common practice. An experiment was, therefore, conducted where one set of test specimens was cured in water where other specimens had been previously stored. A second set of test specimens was placed in fresh water. The aim was to determine whether the water chemistry, i.e., freshwater vs. previously used water, could significantly influence the results of the tests performed by the DiCh-A method. As was subsequently discovered, an important factor that significantly influenced the test results was that each laboratory used water from their local sources.

To investigate this effect, a further large-scale experiment was designed to test whether the content of free CO_2_ in the water storage could statistically significantly affect the test results. In this case, a more extensive set of 150 mm concrete test cubes had already been prepared.

### 3.1. Pilot Experiment—Mortar Testing

The pilot experiment involved 18 test specimens with dimensions 40 × 40 × 160 mm produced from mortar with EN 196-1 standard silica sand [38] and CEM I 42.5 R cement. Each laboratory received six test specimens. Three test specimens were always placed in water where other concrete test specimens had previously been cured. It was, therefore, water which was a saturated solution of calcium hydroxide (used water). The other three specimens were placed in fresh water from their local sources (fresh water).

**Figure 1:**
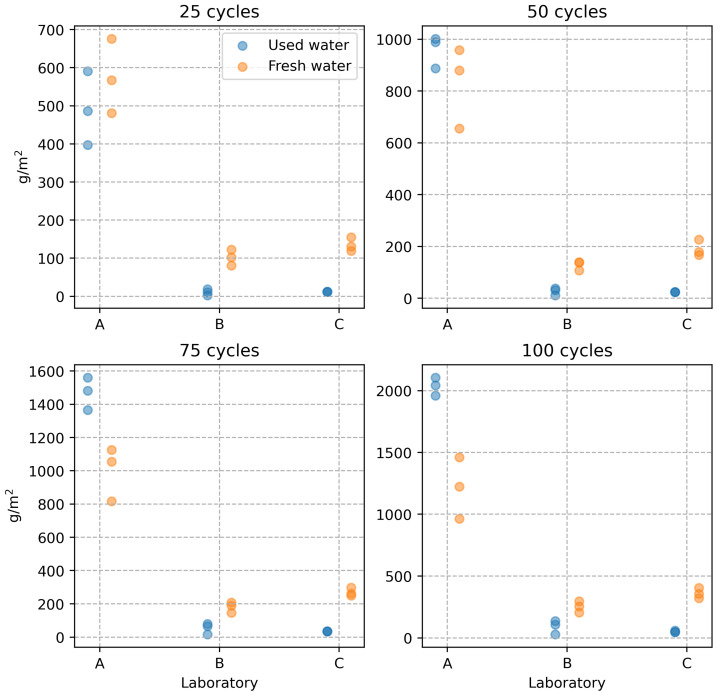
Mortar scaling resistance test results.

The results were statistically processed after performing 100 freeze–thaw cycles according to the DiCh-A method and after determining the amount of scaled off material from the specimen after every 25 cycles. First, the equality of the means of the results of the tests performed by the three laboratories was tested at the 0.05 significance level. The one-factor analysis of variance (ANOVA) was used. The tests were performed separately for each level of freeze–thaw cycles and the water used in the water storage. Based on the analyses performed, the differences between the test results are statistically significant at each experiment level. Therefore, the results of the tests conducted by the three laboratories are statistically significantly different. This is also illustrated in a graphical representation (see Figure 1).

The tests performed by the A-lab show significantly higher values of the scaled off material in g/m^2^ at all experiment levels, i.e., at 25 freeze–thaw cycle intervals. However, there were more interesting observations from the point of view of the objective of the experiment performed. In the design of the experiment, it was assumed that using fresh water to store the test specimens would increase the results of the tests performed according to the DiCh-A method. The results of laboratories B and C showed a slight increase in the amount of scaled off material from the specimens. However, in the case of laboratory A, the effect of the character of the water storage is exactly the opposite. This effect is most evident after 100 freeze–thaw cycles. The average values obtained differ by more than 500 g/m^2^, corresponding to an almost 50% increase in the scaled off material from the specimen surface. By analysing the test results, the authors concluded that the opposite effect in the different laboratories could be caused by the fact that each laboratory used water from their local source to store the test specimens. It was discovered that the DiCh-A method’s test results could be mainly influenced by the content of free CO_2_ in the water used for water storage. As will be shown later in the paper, also based on the results of microscopic analysis, a very thin CaCO_3_ layer forms on the test specimens at higher free CO_2_ content in the water, which leads to a certain hardening of their surface. This hardening does not affect the strength characteristics of the concrete but may affect the results of concrete surface layer tests, such as the amount of scaled off material in the specimen according to the DiCh-A method.

### 3.2. The Second Experiment—Testing Concrete

The significant differences from the pilot experiment were as follows. The experiment was conducted in a proficiency testing programme involving 23 mostly accredited testing laboratories. By participating in similar precision experiments, the laboratories have demonstrated their proficiency in the testing procedures under consideration. The organiser provided nineteen laboratories with three cube-shaped test specimens with an edge of 150 mm. The remaining four laboratories received nine of those test specimens. All 105 test specimens were produced from one batch, at one time, in one place, and using the same mould type. Fresh concrete of class C 30/37 XF4 S4 D${}_{max}$ 16 was made at the concrete plant in a 1 m^3^ mixer. The composition of the fresh concrete is given in Table 1.

**Figure 2:**
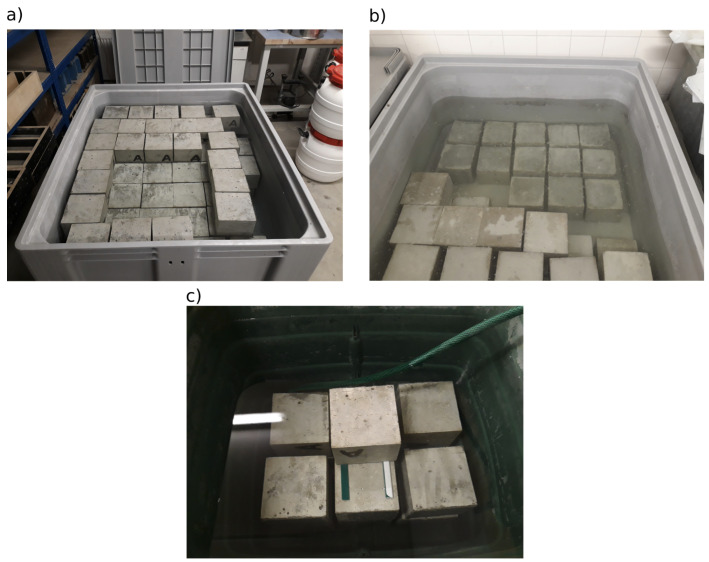
Arrangement of test specimens in (**a**) fresh water, (**b**) previously used water, and (**c**) slow flowing water.

After 24 h from the concrete mixing, the test specimens were de-moulded and placed in different water storage tanks. Sixty-nine specimens were placed in an empty and clean tank that was filled with fresh water from the water supply (Figure 2a). These specimens were used for the main part of the inter-laboratory comparison (hereinafter referred to as “fresh water”). The other 12 test specimens were placed in a tank with previously used water for concrete specimens not intended for this experiment. This water could be considered a saturated solution of calcium hydroxide (Figure 2b), and this storage is referred to as “previously used water”. The remaining 12 specimens were placed in a tank with a constant inflow of water from the water supply (Figure 2c), and this storage is referred to as “slow flowing water”. The water flow rate was adjusted so that saturation with concrete leachates could not occur throughout the maturation process, i.e., for the remaining 27 days. The aim was to maximise the leaching of calcium hydroxide from the test specimens. The differences in water properties in the water storage were also monitored by measuring the pH value: slow flowing water, 6.67; fresh water, 11.44; previously used water, 13.33. The pH measurements were taken with an automatic temperature-compensated probe at 14 days and then 21 days after production. The values are from the second measurement. At 28 days after production, all test specimens were wrapped immediately after removal from water storage in impermeable foil and distributed to the participants of the experiment.

## 4. Results and Discussion

### 4.1. Validation of Test Results

The first step in the analysis of the main part of the experiment was to evaluate the statistical performance of the four laboratories that participated in the main part of the experiment. This was to assess whether the results of the laboratories were statistically significantly different from the results of the tests performed by other laboratories in the large-scale inter-laboratory comparison (ILC). The results of the tests performed by 23 laboratories according to ČSN 73 1326 [36] on specimens under the same conditions were assessed. These specimens were cured in a water storage environment called “fresh water”.

Before the actual assessment of statistical performance using the so-called *z*-score, it was evaluated whether any of the test results could be considered outliers. For this purpose, the Cochran and Grubbs tests were performed at a significance level of 0.01 [39]. Based on exceeding the critical values of the tests, one laboratory was excluded from the experiment due to outlier results at all experiment levels. Due to the large scale of the ILC evaluation, only the outputs after 100 freeze–thaw cycles are presented below.

**Figure 3:**
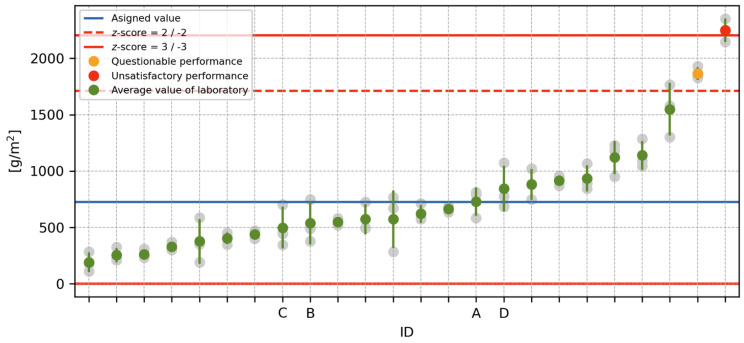
Evaluation of the results of the inter-laboratory comparison.

The mean values and sample standard deviations of the test results of all laboratories are shown in Figure 3. As can be observed from the graph, the performance of one of the laboratories was classified as “unsatisfactory” and that of one laboratory as “problematic” based on the z-score [40]. The assigned value, indicated by the blue line in Figure 3, was evaluated by iterative algorithm A [41]. This algorithm has the advantage of being robust and, thus, less sensitive to outliers than the ordinary arithmetic mean. Threshold values for |z-score|>2 and 3 are shown in Figure 3 in red.

The ILC evaluation also determined a repeatability value of r=50.1% and reproducibility of R=202.6% according to [39]. These values indicate how much variability can be expected between the results of tests performed by one laboratory (repeatability) and multiple laboratories (reproducibility). According to [36], the test procedures show significantly higher *r* and *R* values than other commonly used concrete testing procedures. This is also evident from the results of other ILCs, which can be found, e.g., at [42].

However, the most important outcome of this part of the experiment is that the performance of the laboratories marked A to D, i.e., the laboratories participating in the main part of the experiment, was found to be satisfactory. The resulting *z*-scores even lie in the interval $\langle -1;1\rangle$, indicating that the outputs of these laboratories can be considered satisfactory.

### 4.2. Assessment of Homogeneity of Test Results

**Figure 4:**
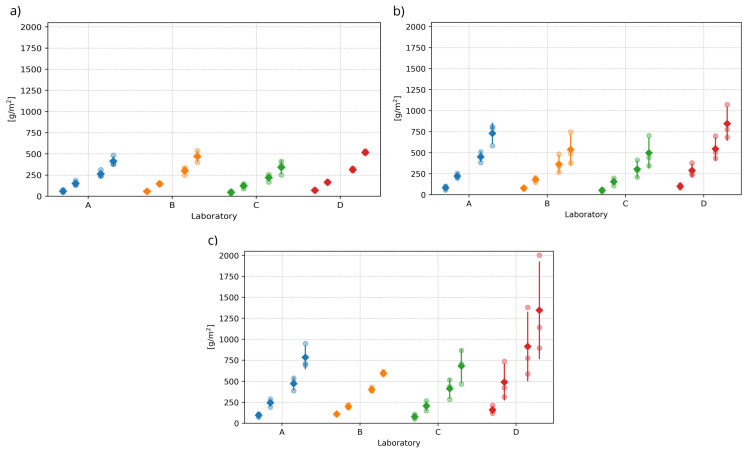
Comparison of test results of the main part of the experiment. The test results of each laboratory are shown for all multiples of freeze–thaw cycles. From the left: 25, 50, 75 and 100 F–T cycles, according to [36]: (**a**) Slow flowing water (**b**) Fresh water (**c**) Previously used water.

**Figure 5:**
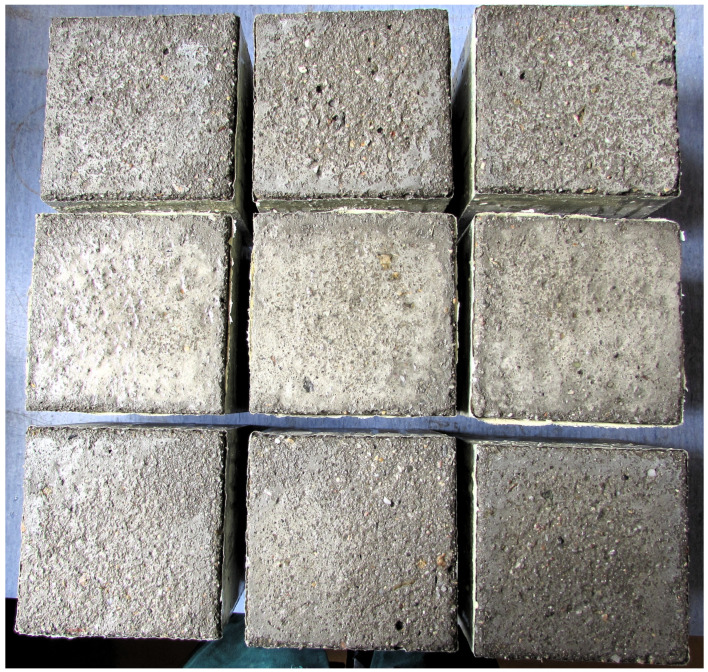
The test specimens of laboratory A after 100 freeze–thaw cycles. At the top are the specimens stored in fresh water, in the middle are the specimens stored in slow flowing water, and at the bottom are the specimens stored in previously used water.

The next phase of the evaluation focused only on the results of laboratories A to D, which measured test specimens stored during curing in water labelled “fresh water” and the other types of water storage described above. The results of the tests, including the means and sample standard deviations, are shown in Figure 4. Figure 4 presents the test specimens of laboratory A after 100 freeze–thaw cycles. It is noticeable from the surface colouration that the surface has been damaged differently by de-icing chemicals (see Figure 5).

**Figure 6:**
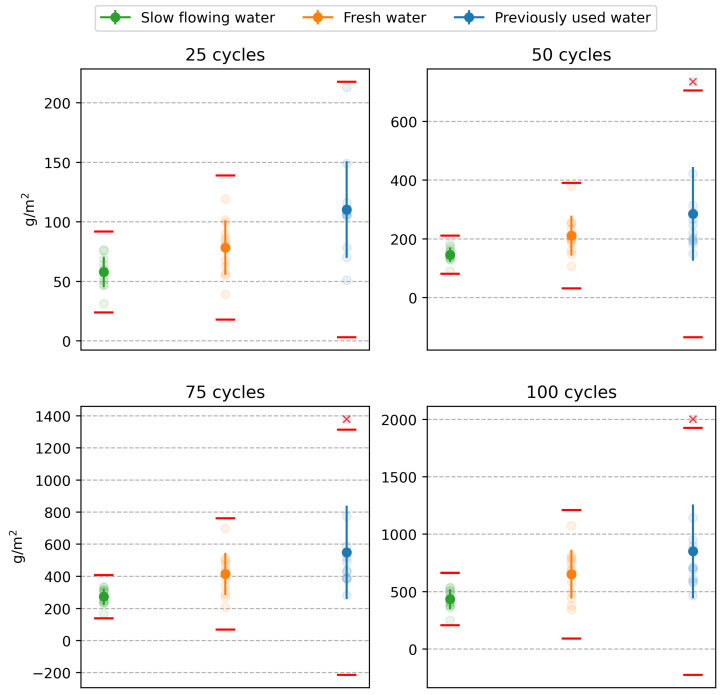
Test results: the red lines represent the 1% critical limit of the Grubbs test, the red crosses represent outliers.

**Figure 7:**
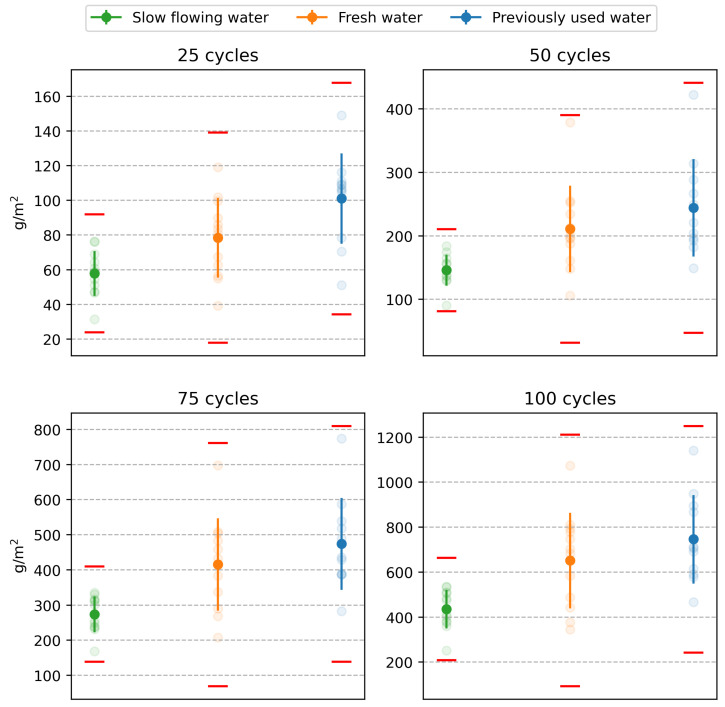
Test results after elimination of outliers.

The first step in the statistical analysis of the results of laboratories A to D was to consider whether the differences between the individual test results exhibit statistically significant differences. The aim was to determine whether the results of the tests conducted each time on specimens from the same water storage can be mixed and, thus, be considered as a homogeneous statistical set suitable for further evaluation. At the same time, these tests will demonstrate whether the experimental outputs can be influenced by a higher level of reproducibility (see above). The so-called analysis of variance (ANOVA) was applied, whose null hypothesis is the equality of means of the results between laboratories. Testing was performed at the 0.01 level of significance. The results of the ANOVA tests, presented as *p*-values in Table 2, indicate no statistically significant differences between the results of the different laboratories. At the same time, the red cross in Figure 6 indicates individual test results that were flagged as outliers by the Grubbs test after mixing the values and were, therefore, excluded from the experiment. A graphical representation of all non-outlying results is shown in Figure 7. The test results of one specimen that showed a significantly higher amount of scaled off material from the specimen after 50, 75, and 100 freeze–thaw cycles were excluded.

Table 2 also shows the results of the normality tests performed by the Shapiro–Wilk test. As can be seen, normality was accepted at the 0.05 level of significance for the results of the tests of the specimens stored in the fresh and slow flowing water. The results for the test specimens stored in previously used water showed normality only after outliers were removed. Since no results were discarded for the first two storage conditions, there was no reason to retest for normality. The confirmation of normality shows, among other factors, that the test results are unaffected by any systematic factors and that merging results across laboratories was justified.

The next step in the analysis was to determine whether the character of the water in the storage significantly influenced the test results. For this purpose, the analysis of variance (ANOVA) was again applied. This time, the combined test results across laboratories A to D were tested in a single statistical set. The results of these tests, in the form of *p*-values, are presented in Table 3. Since all *p*-values show values below the pre-specified significance level of 0.01, it can be concluded that the results are statistically significantly different. This indicates that the nature of the water in the water storage significantly affects the results of the DiCh-A method tests.

### 4.3. Assessment of Test Results from the Perspective of Chemical Processes

**Figure 8:**
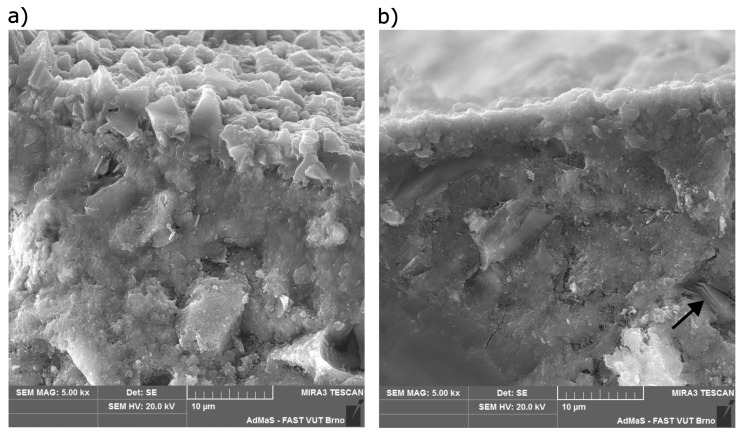
(**a**) Cross-sectional image of a specimen stored in slow flowing water. (**b**) Cross-sectional image of a specimen stored in previously used water.

The most significant differences can be observed between the tests performed on specimens placed in slow flowing water and previously used water. Scanning electron microscope (SEM) images of specimens from these water storages are shown in Figure 8. Part (a) shows an image of a fraction of a concrete specimen stored in slow flowing water. It is evident that the surface of the concrete is covered with a continuous layer of calcium carbonate (CaCO_3_) crystals about 5 to 7 µm thick, while only isolated CaCO_3_ crystals were found on the surface of the concrete stored in the previously used water (see Figure 8b). These test specimens contained portlandite Ca(OH)_2_ crystals near the surface, as indicated by the black arrow in Figure 8. No portlandite crystals were found in concrete placed in slow flowing water at the same depth from the surface. The identification of the different crystal phases was performed with an EDX probe.

It can be concluded that calcium hydroxide dissolves in the flowing water and reacts with the carbon dioxide contained in the water to form calcite (CaCO_3_). An important factor is the content of aggressive CO_2_ and bicarbonate in the water. Calcium and magnesium bicarbonates cause a transient hardness of the water, and their solubility depends on the CO_2_ content of the water. If the amount of CO_2_ in the water decreases, the bicarbonates decompose to exclude the insoluble carbonates (CaCO_3_ and MgCO_3_), which results in a decrease in water hardness. Conversely, when the carbon dioxide content of the water increases, CaCO_3_ is progressively transferred into solution according to the equation:

(1) $$\mathrm{CaC}O_{3}+H_{2}O+CO_{2}⇌Ca^{2+}+2\mathrm{HC}O_{3}^{-}.$$

The released CaCO_3_ creates a thin, tightly attached layer on the concrete surface of the test specimens and significantly contributes to the durability of the entire concrete surface layer. This effect was observed during the curing of the specimens in the described experiment and is confirmed by the scanning electron microscope (SEM) images; see Figure 8. The results of the experiment thus show that the amount of scaled off material detected from a specimen during the de-icing chemicals resistance test (according to the DiCh-A method) depends not only on the quality of the concrete under testing but also on the nature of the water in which the specimens have been stored before testing.

The analysis of the tap water by Heyer’s test showed an aggressive CO_2_ concentration of 12.76 mg/L and a Langelier index of −0.14. Thus, this is indeed water with a high aggressive CO_2_ content.

It would certainly be interesting to also find out whether the thin, firmly adhering CaCO_3_ layer on the surface of the test specimens does not significantly influence the results of other test methods verifying the quality of the concrete surface layer, such as the ISAT [43], GWT [44], TPT [45], or carbonation resistance test [46,47].

## 5. Conclusions

Based on the conducted experiments, the following can be stated:

- The aggressive CO_2_ and bicarbonate content in water storage is an important factor influencing the formation of CaCO_3_ on the concrete surface.
- The leached Ca(OH)_2_ from the concrete into the water leads to the production of CaCO_3_, which forms a thin, firmly adhering layer on the concrete surface that significantly contributes to the peel resistance of the concrete surface layer. This was validated using the ANOVA statistical test at the 0.01 significance level.
- The amount of scaling detected in the de-icing chemicals test depends on the concrete quality and the type of water in which the specimens are stored before testing.
- Further research is needed to better understand the effect of CaCO_3_ and other factors on the durability and quality of the concrete surface layer, especially concerning concrete durability test results.

## Figures and Tables

**Table 1:** The composition of fresh concrete.

Component	kg/m^3^
Cement CEM I 42.5 R (Morká cement plant, CZ)	400
Fine aggregate 0–4 mm (Bratčice, CZ)	807
Coarse aggregate 8–16 mm (Olbramovice, CZ)	915
Air-entraining admixture LPS A 94 (Sika, CZ)	1
Plasticizing admixture SVC-4035 (Sika, CZ)	2.2
Water	182

**Table 2:** Results of statistical tests.

Water Storage	Number of Cycles	ANOVA *p*-Value	Shapiro Test *p*-Value (Normality Test)	Shapiro Test *p*-Value (Normality Test) after Excluding Outliers
Slow flowing water	25	0.141	0.810	-
	50	0.169	0.633	-
	75	0.065	0.174	-
	100	0.038	0.262	-
Fresh water	25	0.043	0.992	-
	50	0.047	0.227	-
	75	0.099	0.861	-
	100	0.131	0.647	-
Previously used water	25	0.052	0.044	0.204
	50	0.045	0.001	0.232
	75	0.061	0.001	0.362
	100	0.072	0.003	0.654

**Table 3:** Results of the ANOVA tests between types of water storage.

Number of Cycles	*p*-Value
25	0.000141
50	0.001576
75	0.000399
100	0.000480

## Data Availability

The data presented in this study are available on request from the corresponding author.

## References

[1] Prasad Yadav K., Samanta A.K. (2023). Characterization and Development of Alccofine Based Sustainable Concrete—A Review. Mater. Today Proc..

[2] Sharma D., Sharma S., Goyal A. (2016). Utilization of Waste Foundry Slag and Alccofine for Developing High Strength Concrete. Int. J. Electrochem. Sci..

[3] U.S.: Cement Production 2022. https://www.statista.com/statistics/219343/cement-production-worldwide/.

[4] Monteiro P.J.M., Miller S.A., Horvath A. (2017). Towards Sustainable Concrete. Nat. Mater..

[5] (2018). Making Concrete Change: Innovation in Low-Carbon Cement and Concrete. https://www.chathamhouse.org/2018/06/making-concrete-change-innovation-low-carbon-cement-and-concrete.

[6] Wada Y., Van Beek L.P.H., Van Kempen C.M., Reckman J.W.T.M., Vasak S., Bierkens M.F.P. (2010). Global Depletion of Groundwater Resources: GLOBAL GROUNDWATER DEPLETION. Geophys. Res. Lett..

[7] Nilimaa J. (2023). Smart Materials and Technologies for Sustainable Concrete Construction. Dev. Built Environ..

[8] Grebenkov D.S. (2022). Depletion of Resources by a Population of Diffusing Species. Phys. Rev. E.

[9] Müller H.S., Haist M., Vogel M. (2014). Assessment of the Sustainability Potential of Concrete and Concrete Structures Considering Their Environmental Impact, Performance and Lifetime. Constr. Build. Mater..

[10] Alexander M.G., Bentur A., Mindess S. (2017). Durability of Concrete: Design and Construction.

[11] Kocáb D., Terzijski I., Strnad J., Ševčík M. (2020). Determination of Concrete Resistance to Corrosive Effects of Water with Aggressive CO_2_ Using Model Mortars. Solid State Phenom..

[12] Terzijski I., Kocáb D., Štěpánek P., Strnad J., Girgle F., Šimůnek P. (2021). Development of Variants of High-Performance Self-Compacting Concrete with Improved Resistance to the Attack of Sulfates. Appl. Sci..

[13] Piasta W., Góra J., Turkiewicz T. (2016). Properties and Durability of Coarse Igneous Rock Aggregates and Concretes. Constr. Build. Mater..

[14] Collepardi M. (2006). The New Concrete.

[15] Yi Y., Zhu D., Guo S., Zhang Z., Shi C. (2020). A Review on the Deterioration and Approaches to Enhance the Durability of Concrete in the Marine Environment. Cem. Concr. Compos..

[16] Ma D., Zhang M., Cui J. (2023). A Review on the Deterioration of Mechanical and Durability Performance of Marine-Concrete under the Scouring Action. J. Build. Eng..

[17] Maus S., Bahafid S., Hendriks M., Jacobsen S., Geiker M.R. (2023). X-ray Micro-Tomographic Imaging and Modelling of Saline Ice Properties in Concrete Frost Salt Scaling Experiments. Cold Reg. Sci. Technol..

[18] Nowak-Michta A. (2022). Salt Scaling Resistance of Variable w/c Ratio Air-Entrained Concretes Modified with Polycarboxylates as a Proper Consequence of Air Void System. Materials.

[19] Faheem A., Hasholt M.T. (2022). The Effect of Temperature Distribution in Mortar on Frost Scaling. Cem. Concr. Res..

[20] Zhang J., Wang J., Li Y., Yuan J., Wu Y. (2022). Research Progresses on Salt Scaling and Protective Methods for Concrete Pavements. Constr. Build. Mater..

[21] Ding Z., Quy N.X., Kim J., Hama Y. (2022). Evaluations of Frost and Scaling Resistance of Fly Ash Concrete in Terms of Changes in Water Absorption and Pore Structure under the Accelerated Carbonation Conditions. Constr. Build. Mater..

[22] Cappellesso V.G., Van Mullem T., Gruyaert E., Van Tittelboom K., De Belie N. (2023). Bacteria-Based Self-Healing Concrete Exposed to Frost Salt Scaling. Cem. Concr. Compos..

[23] Liu Z., Hansen W. (2015). Freezing Characteristics of Air-Entrained Concrete in the Presence of Deicing Salt. Cem. Concr. Res..

[24] Valenza J.J., Scherer G.W. (2007). Mechanism for Salt Scaling of a Cementitious Surface. Mater. Struct..

[25] Valenza J.J., Scherer G.W. (2007). A Review of Salt Scaling: I. Phenomenology. Cem. Concr. Res..

[26] Fagerlund G., Setzer M. (1992). Freeze-Thaw and de-Icing Resistance of Concrete: Research Seminar Held in Lund, June 17, 1991: RILEM Committee TC-117 FDC. Report TVBM.

[27] Shang H.S., Cao W.Q., Wang B. (2014). Effect of Fast Freeze-Thaw Cycles on Mechanical Properties of Ordinary-Air-Entrained Concrete. Sci. World J..

[28] Penttala V. (2006). Surface and Internal Deterioration of Concrete Due to Saline and Non-Saline Freeze–Thaw Loads. Cem. Concr. Res..

[29] Sun Z., Scherer G.W. (2010). Effect of Air Voids on Salt Scaling and Internal Freezing. Cem. Concr. Res..

[30] Kocáb D., Misák P., Vymazal T., Komárková T., Halamová R. (2017). Stanovení odolnosti povrchu betonu proti působení vody a chemických rozmrazovacích látek—Metody, praxe a problémy. Beton TKS.

[31] (2016). Testing Hardened Concrete—Part 9: Freeze-Thaw Resistance with De-Icing Salts—Scaling.

[32] (2005). Betongprovning—Hårdnad Betong—Draghållfasthet Hos Provkroppar.

[33] RILEM TC 117-FDC (1996). TC 117-FDC Recommendation—CDF Test—Test Method for the Freez Thaw and Deicing Resistance of Concrete—Tests with Sodium Chloride (CDF). Mater. Struct..

[34] (2012). Standard Test Method for Scaling Resistance of Concrete Surfaces Exposed to Deicing Chemicals.

[35] Vassilev D.G. (2012). Evaluation of Test Methods for De-Icer Scaling Resistance of Concrete. Ph.D. Thesis.

[36] ČSN 73 1326: Stanovení Odolnosti Povrchu Cementového Betonu Proti Působení Vody a Chemických Rozmrazovacích Látek, 1985. ČSN 73 1326. http://seznamcsn.agentura-cas.cz/Detailnormy.aspx?k=31089.

[37] Kocáb D., Komárková T., Králíková M., Misák P., Moravcová B. (2017). Experimental Determination of the Influence of Fresh Concrete’s Composition on Its Resistance to Water and de-Icing Chemicals by Means of Two Methods. Mater. Tehnol..

[38] (2016). Methods of Testing Cement—Part 1: Determination of Strength.

[39] (2023). Conformity assessment – General requirements for proficiency testing.

[40] (2019). Accuracy (Trueness and Precision) of Measurement Methods and Results—Part 2: Basic Method for the Determination of Repeatability and Reproducibility of a Standard Measurement Method.

[41] (2022). Statistical Methods for Use in Proficiency Testing by Interlaboratory Comparison.

[42] MPZ. http://www.ptprovider.cz/.

[43] (2020). Testing Concrete. Recommendations for the Determination of the Initial Surface Absorption of Concrete.

[44] Moczko A., Moczko M. (2016). GWT—New Testing System for “in-Situ” Measurements of Concrete Water Permeability. Procedia Eng..

[45] Zhang D., Li K. (2019). Concrete Gas Permeability from Different Methods: Correlation Analysis. Cem. Concr. Compos..

[46] (2018). Testing Hardened Concrete—Part 10: Determination of the Carbonation Resistance of Concrete at Atmospheric Levels of Carbon Dioxide.

[47] (2020). Testing Hardened Concrete—Part 12: Determination of the Carbonation Resistance of Concrete—Accelerated Carbonation Method.

